# Supplementary material for: Conventional Cancer Therapies Can Accelerate Malignant Potential of Cancer Cells by Activating Cancer-Associated Fibroblasts in Esophageal Cancer Models
Source: Cancers (Basel). 2023 May 30;15(11):2971. doi: 10.3390/cancers15112971 (PMC10252052; doi:10.3390/cancers15112971)
Supplement: Supplementary file 1 [file cancers-15-02971-s001.zip › cancers-2353408-supplementary.pdf]

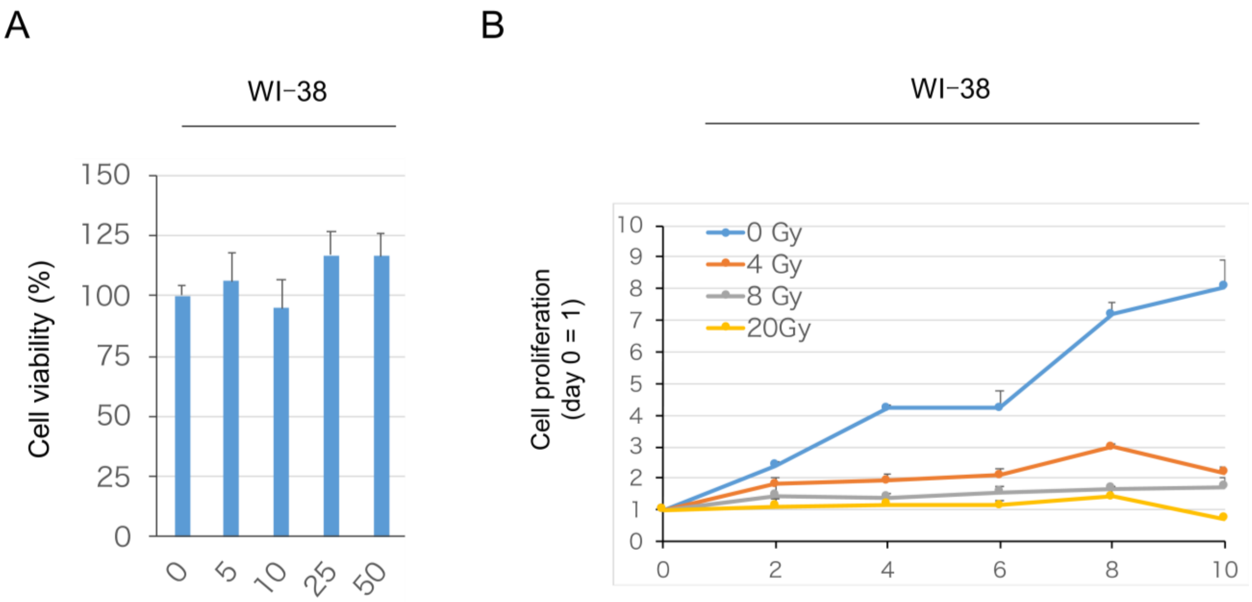

Figure S1. Efficacy of radiotherapy against WI-38 fibroblasts. (A) Viability of WI-38 cells induced by radiotherapy measured using XTT (n = 4. Mean ± SD). The representative example from three experiments was shown. (B) Relative proliferation curves of WI-38 cells treated by radiotherapy. Day 0 is set as the control (n = 4; mean ± SD). The representative example from three experiments was shown.

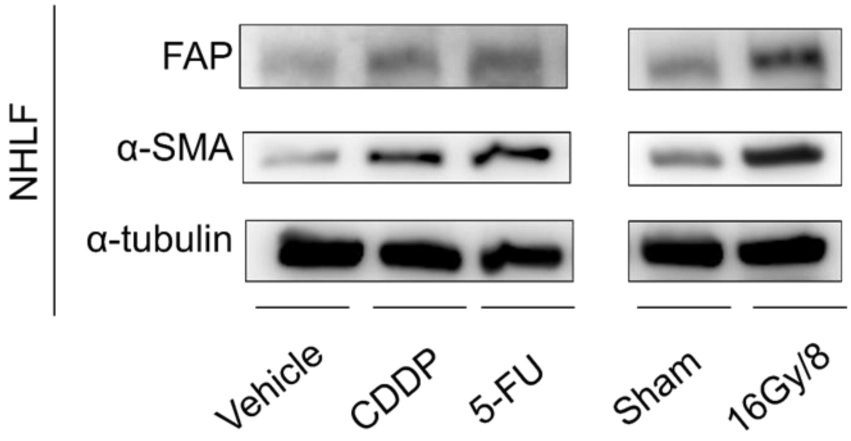

Figure S2. Western Blotting for αSMA and FAP in fibroblasts. Fibroblast cells were lysed using cell lysis buffer (50 mmol/L Tris-HCl (pH 7.4), 30 mmol/L NaCl, and 1% Triton X-100) containing protease inhibitors (Complete Mini, Roche Diagnostics GmbH, Basel, Switzerland). Proteins extracted from whole-cell lysates were electrophoresed on polyacrylamide gels and transferred onto Hybond-polyvinylidene difluoride transfer membranes (GE Healthcare, UK). The membranes were incubated with primary antibodies against FAP (#M01,

Abnova, Taiwan), αSMA (ab8211, Abcam, UK), or α-tublin (#2144, Cell Signaling Technology, MA, US), overnight at 4 °C, followed by secondary antibodies for 1 hour at room temperature. The membranes were visualized using an LAS-4000 mini (FUJIFILM, Tokyo, Japan). Western blots showed higher expression of FAP and αSMA in treated fibroblasts vs. normal fibroblasts.

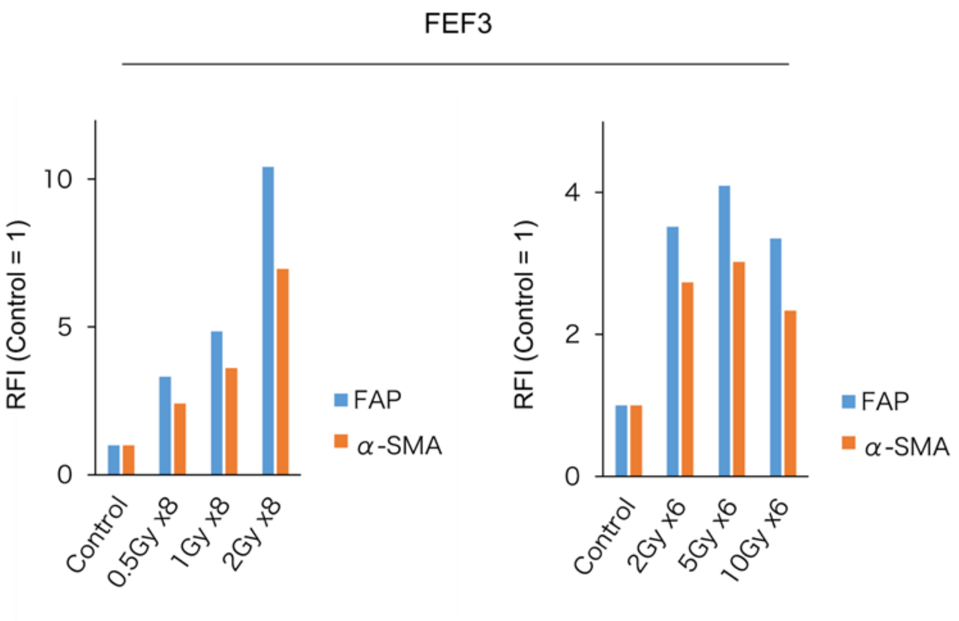

Figure S3. FAP and αSMA expression in treated fibroblasts after various X-ray irradiation. The expressions of FAP or αSMA by flow cytometry in three fibroblasts cells were evaluated. RFI was calculated. The Control is 0 Gy Xray irradiation. The X-axis legends indicate the dose of one irradiation (Gy) × number of irradiations (fx).

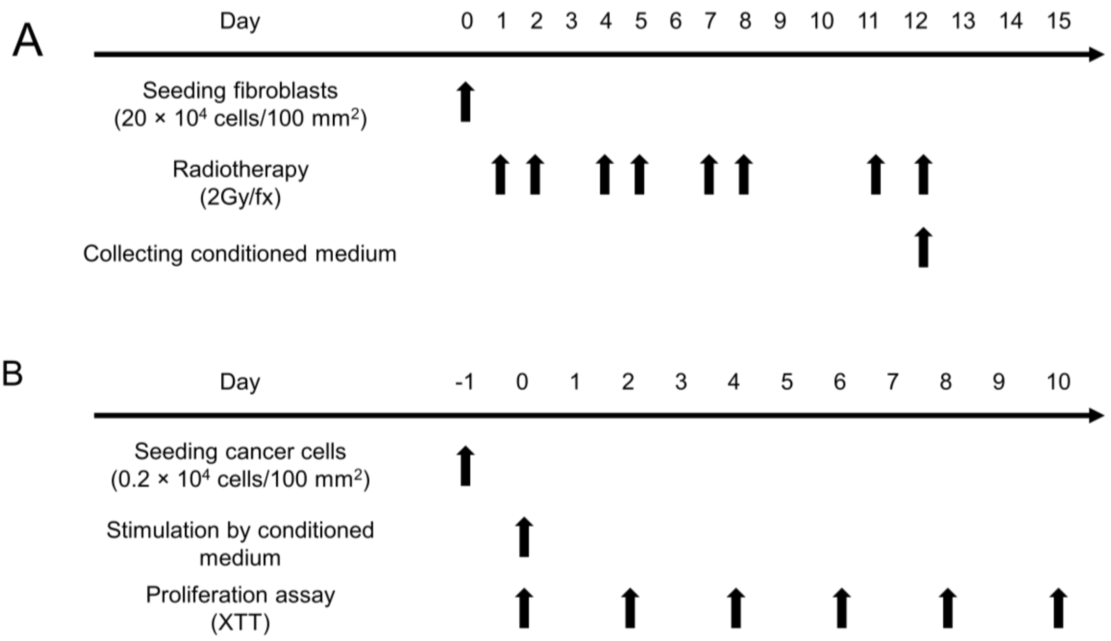

Figure S4. Treatment regimens for XTT assay after treated conditioned medium. (A) Treatment regimen to collect conditioned medium of fibroblasts treated by radiotherapy. (B) Treatment regimen to evaluate metabolic cell viability by XTT using conditioned medium.

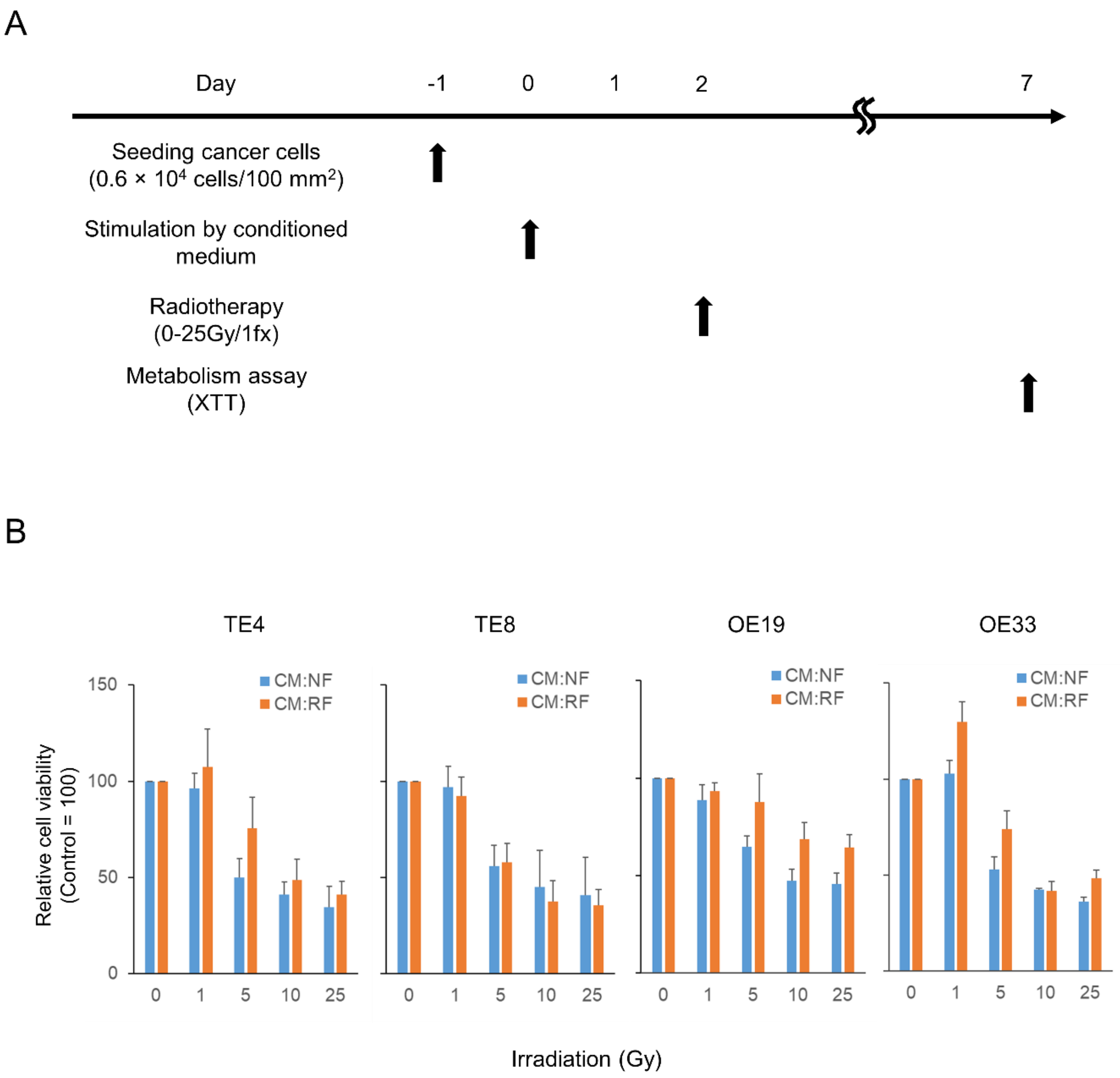

Figure S5 Cell viability in cancer cells treated by conditioned medium after radiotherapy. (A) Treatment regimen. (B) Cell viability of treated esophageal cancer cells induced by radiotherapy was measured by XTT (n = 4. Mean ± SD). CM-NF, conditioned media made from normal fibroblasts; CM-RF, conditioned media made from radiation-treated fibroblasts.

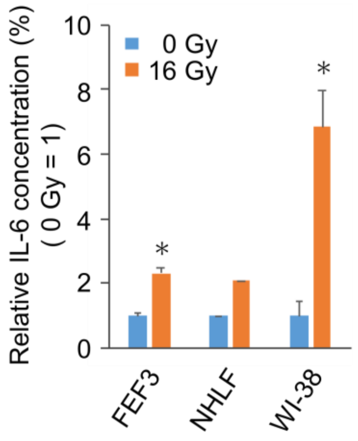

Figure S6. Quantification of IL-6 secretion by human-derived fibroblasts after X-ray irradiation was investigated via ELISA. The level of IL-6 in each supernatant of conditioned media was measured using Quantikine ELISA human IL6 Immunoassay (R & D systems). Each data was acquired as IL-6 levels per 1.0×10^4 cells, then relative IL-6 concentration was calculated. The untreated group (0 Gy) was set as the control group (n =4, Mean ± SD, unpaired t test; *, $P < 0.05$).

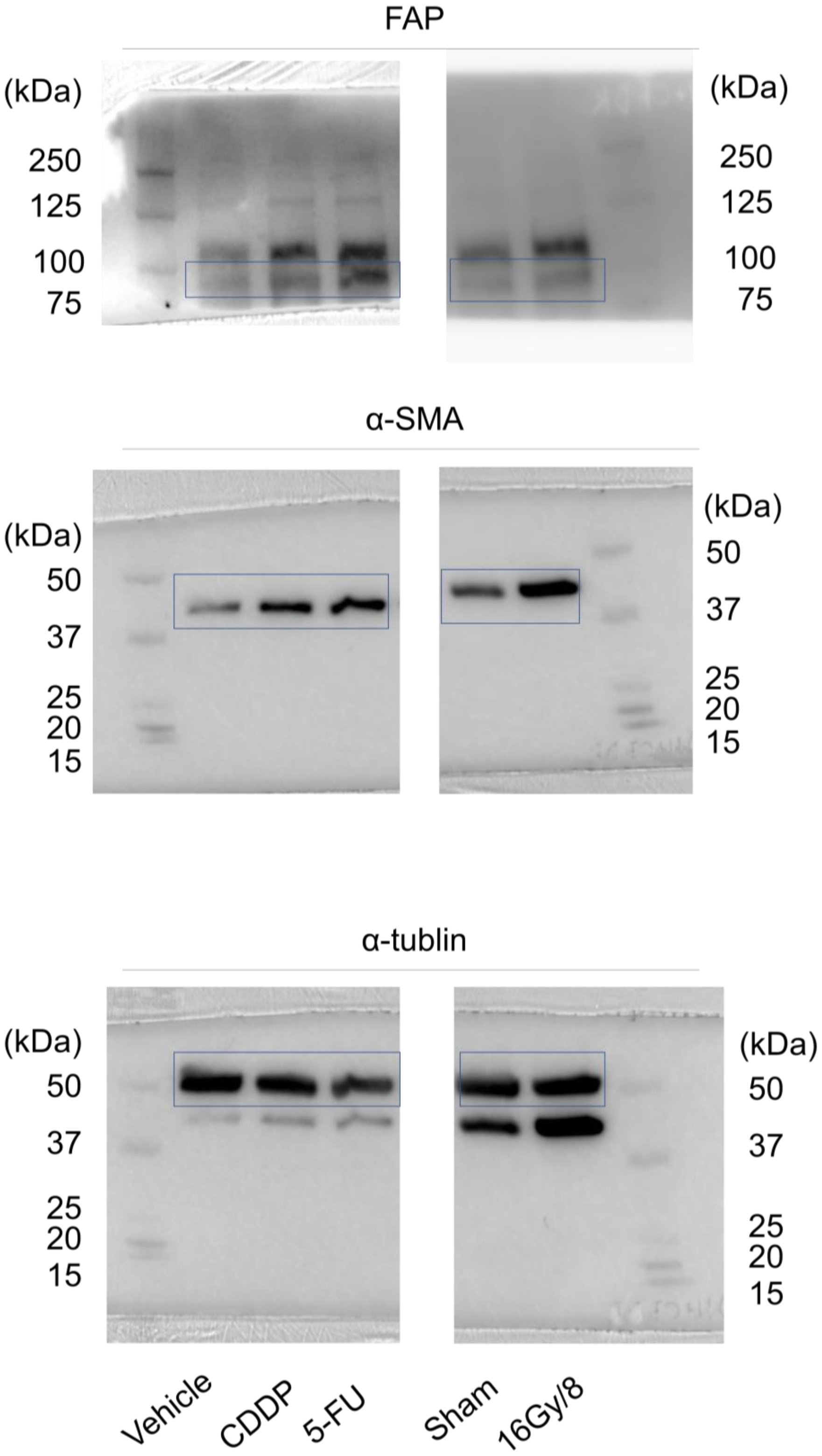

Figure S7. Supplementary information of multiple exposed images of original blots with molecular size markings.
